# Empyema Necessitans: A Rare Complication of Chest Drains

**DOI:** 10.7759/cureus.69931

**Published:** 2024-09-22

**Authors:** Haider Bokhary, Shiza Chaudhry, Abhinav P Kanigicherla, Kanwal Tariq, Michael R Gooseman

**Affiliations:** 1 Internal Medicine, Hull University Teaching Hospital, Hull, GBR; 2 Respiratory Medicine, Hull University Teaching Hospital, Hull, GBR; 3 Thoracic Surgery, Hull University Teaching Hospital, Hull, GBR

**Keywords:** chest drain, decortication, empyema, empyema necessitans, empyema necessitatis, para-pneumonic effusion, pleural effusion, pleural infection, pleural interventions, pneumonia

## Abstract

Empyema is defined as an accumulation of frank pus within the pleural cavity. Empyema necessitans is a rare complication of the pleural space infection when the existing empyema extends into adjacent structures beyond the pleural space, usually into the soft tissue of the chest wall. If severe, it can externalize to the skin. Historically, one of the most common causes of this condition was uncontrolled tuberculous infections.

Our study describes a case where empyema necessitans could be classed as iatrogenic. This patient was initially admitted for empyema secondary to community-acquired pneumonia, which was successfully treated with a course of antibiotics and insertion of a chest drain. She was discharged but had to be re-admitted after one month, this time with a chest wall swelling at the point of recent chest drain insertion, which began to ooze purulent discharge. Imaging confirmed the diagnosis of empyema necessitans, which was then treated with a combination of surgical and antimicrobial therapy.

Empyema necessitans are now seldom encountered, owing mainly to effective antibiotics and anti-tuberculous treatment. It is perhaps even more rarely seen as a complication of chest drain insertion. However, the report emphasizes the need to consider empyema necessitates in the list of differentials when assessing a patient with chest wall swelling, particularly where they have recently undergone pleural intervention.

## Introduction

Empyema is an accumulation of frank pus within the pleural space of the lung caused by either pyogenic inoculations or ongoing pneumonia in a patient. Sometimes, one can encounter a similar variation of empyema called lung abscess, which is characterized by localized collection of pus within the lung parenchyma [1]. Empyema necessitans is a rare complication of the pleural space infection when the existing empyema extends into adjacent structures beyond the pleural space [2]. The most commonly involved structure is the thoracic wall; however, it can involve any adjacent structure such as breast tissue, pericardium, or the peritoneum. On rare occasions, patients can develop this condition as a complication of pleural intervention or trauma causing pus to track through the site of intervention [3].

The most common causes for this condition remain *Mycobacterium tuberculosis* and *Actinomyces *spp. [4]. The overall incidence of this condition has reduced with the advent of effective antibiotic and antituberculous therapy, along with early drainage, to the point where it is now rarely encountered in clinical practice [2]. The diagnosis is best confirmed by computed tomography (CT) imaging and, at times, can determine the prognosis of the patient [5].

## Case presentation

We present a case about an elderly lady who was admitted via the emergency department. She had a one-week history of cough which was productive of thick, green sputum. This was associated with gradually progressive shortness of breath on exertion. At the point of admission, she was managing to walk approximately 100 yards before needing to stop due to breathlessness. She also had right-sided chest pain for two days prior to presenting. This pain was assessed to be pleuritic in character, which localized to the lateral aspect of the right lower chest. The pain was constant, with a severity of 6/10, and was getting worse till the point of admission. Along with her respiratory symptoms, she experienced lethargy, fever with rigors and chills, and reduced oral intake. There was no hemoptysis. There were no other significant symptoms on systemic review.

On examination of the chest, she was noted to have coarse crackles and reduced breath sounds in the right base. There was no stridor or wheeze. The right base had a dull note on percussion. Her heart sounds were normal. There was no abdominal or chest wall tenderness. There was no clubbing or discoloration of her fingernails, no joint deformities, and no signs suggestive of heart failure. On admission, she was initially hypoxic with an oxygen saturation of 86%, which improved to 94% with 2 liters of oxygen via a nasal cannula. Her respiratory rate was 21 per minute and her heart rate was 112 bpm, with a blood pressure of 113/75 mmHg, and she was afebrile. There was no confusion or reduced consciousness at any point throughout the admission.

**Figure 1:**
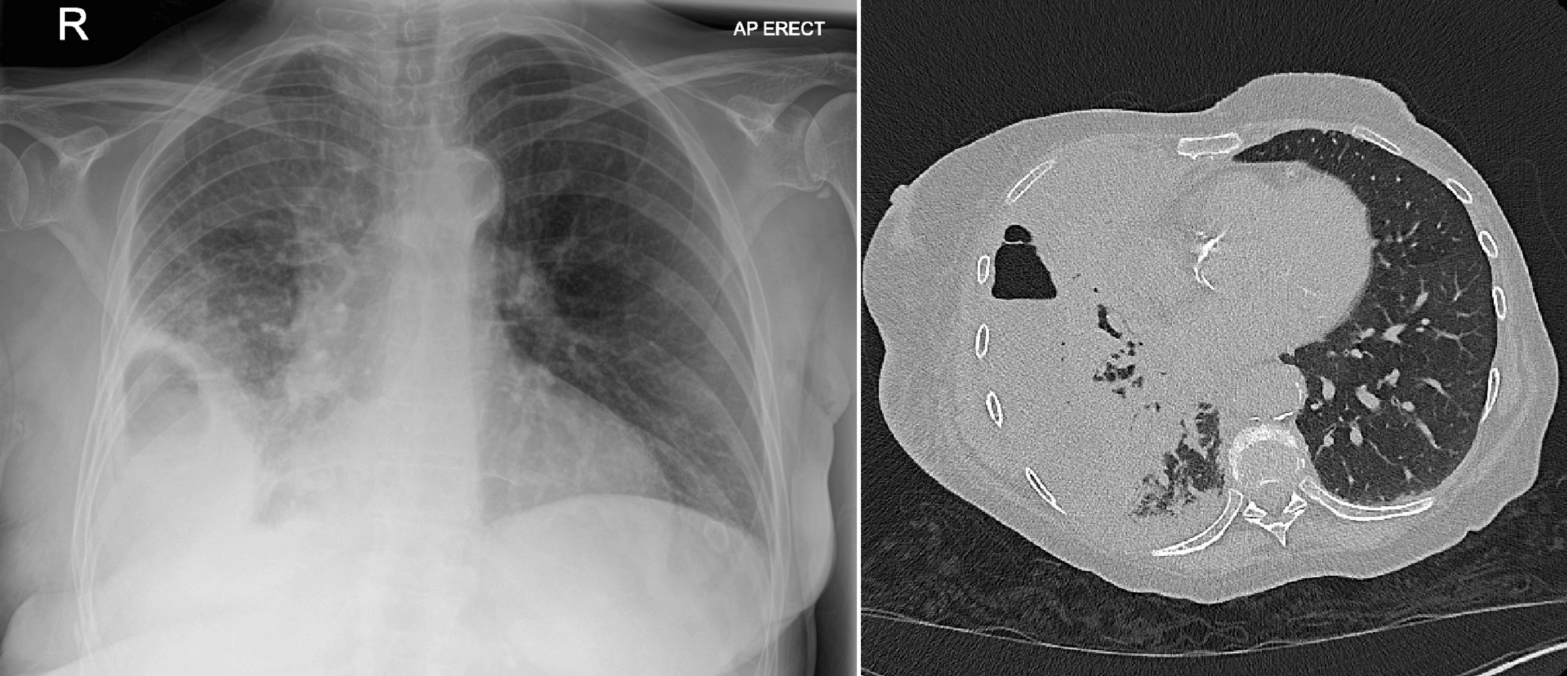
Toward the left side, the chest radiograph at the time of admission. Toward the right side, a cross-sectional image of the CT scan

Blood tests on admission showed a C-reactive protein (CRP) level of 480 mg/L and a white blood count of 29.4 (x10^9/L). The rest of her full blood count, liver function, urea, and electrolytes were all within normal range. Chest X-ray on admission showed shadowing of the right lower to mid zones with a right-sided pleural effusion. To further assess the lung parenchyma and to help plan for pleural intervention, a CT scan of the thorax was performed. This reported a multiloculated right-sided effusion with multiple gas foci in keeping with empyema and overlying consolidation. Pictures of the chest radiography and CT imaging on the first admission are shown in Figure 1.

**Figure 2:**
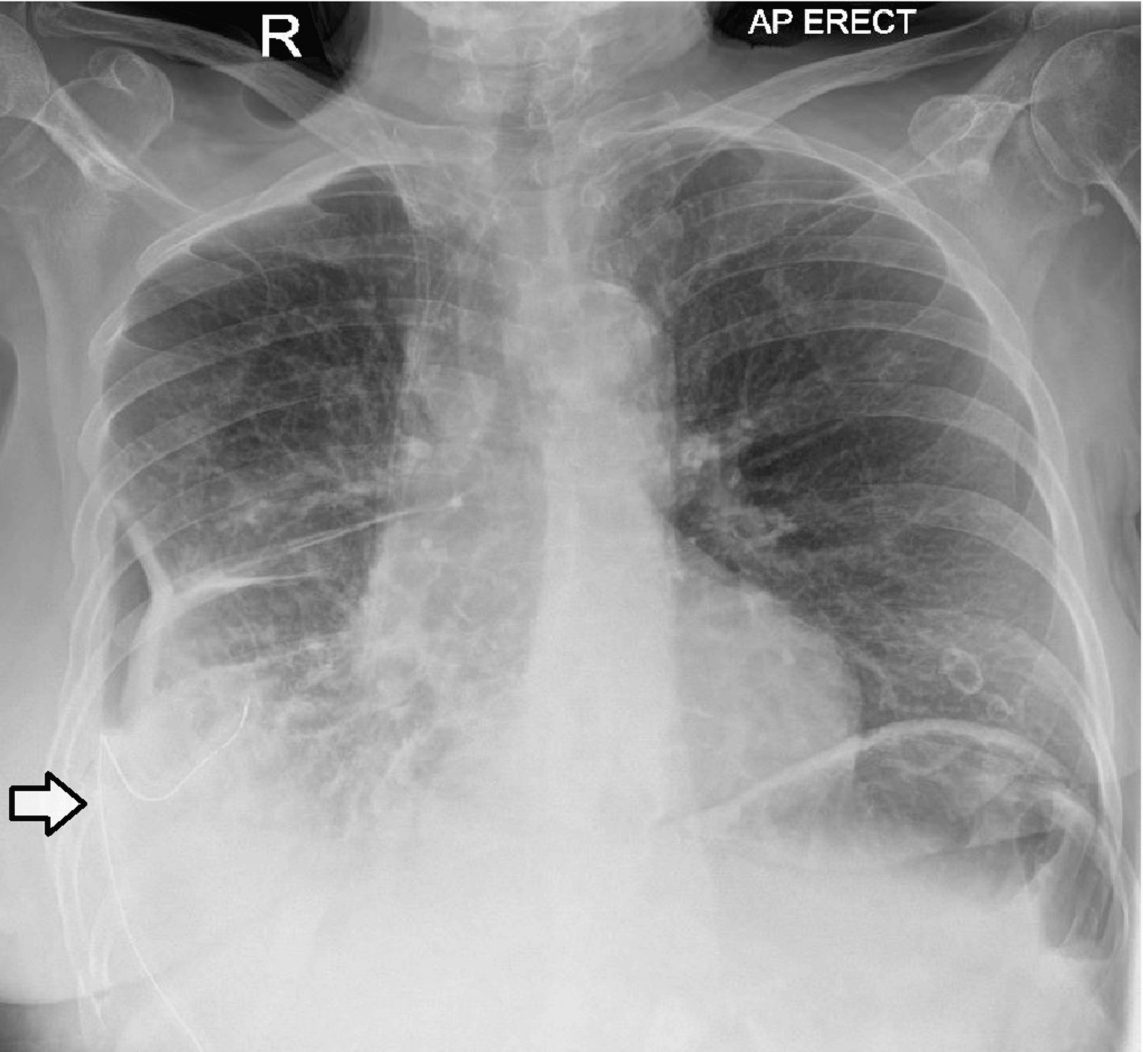
Chest X-ray following chest drain insertion

Treatment was started with intravenous cefuroxime 750 mg three times a day and intravenous metronidazole 500 mg three times a day. An 18-french intercostal chest drain was inserted, which drained frank pus with a pH of 7.05. Pleural fluid samples were sent for microscopy and cultures. Microscopy revealed multiple leukocytes. Pleural fluid culture reported heavy growths of *Streptococcus constellatus* (sensitive to cefuroxime, all penicillins, and clindamycin) and *Parvimonas micra *(sensitive to metronidazole). Pleural fluid acid-fast bacilli and cultures for *Mycobacterium *were negative. A chest X-ray following chest drain insertion is shown in Figure 2.

**Figure 3:**
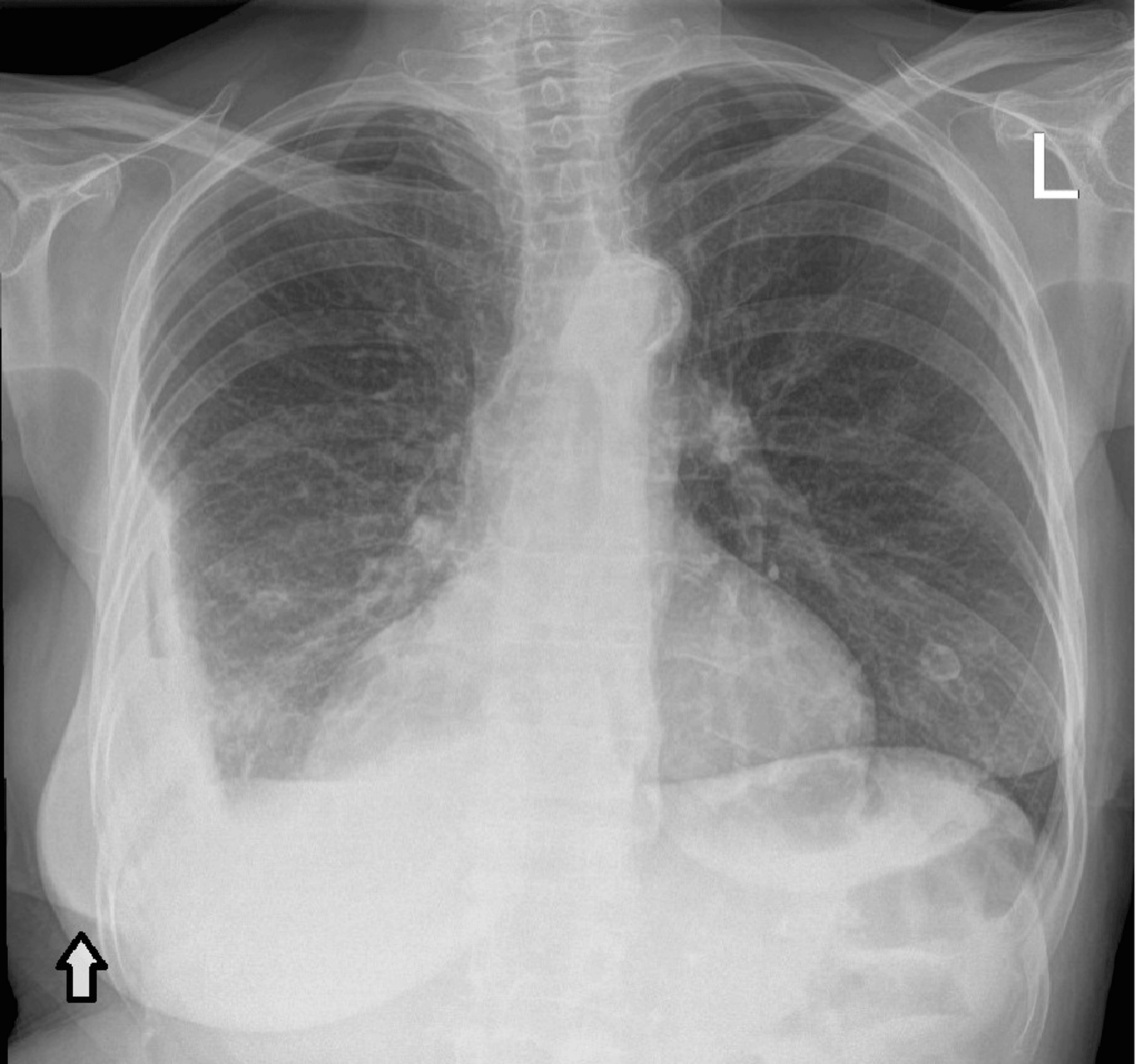
Chest X-ray following drain removal. Note the extension of the empyema into the thoracic wall

She completed a one-week course of intravenous cefuroxime and metronidazole. As per sensitivities of the pleural fluid, the antibiotics were changed to per oral amoxicillin and metronidazole. The chest drain remained in situ for a total of 12 days; it was regularly flushed and drained well until the point of removal. Clinically, she had responded very well to the treatment received. Her blood markers had improved; CRP had come down to 62 mg/L and her white blood count reduced to 16.1 (x10^9/L). A chest radiograph at the time of discharge showed marked improvement in the size of the effusion. However, as labeled on the following imaging, a tract could be seen developing at the drain site. The chest X-ray done four hours after chest drain removal is shown in Figure 3.

**Figure 4:**
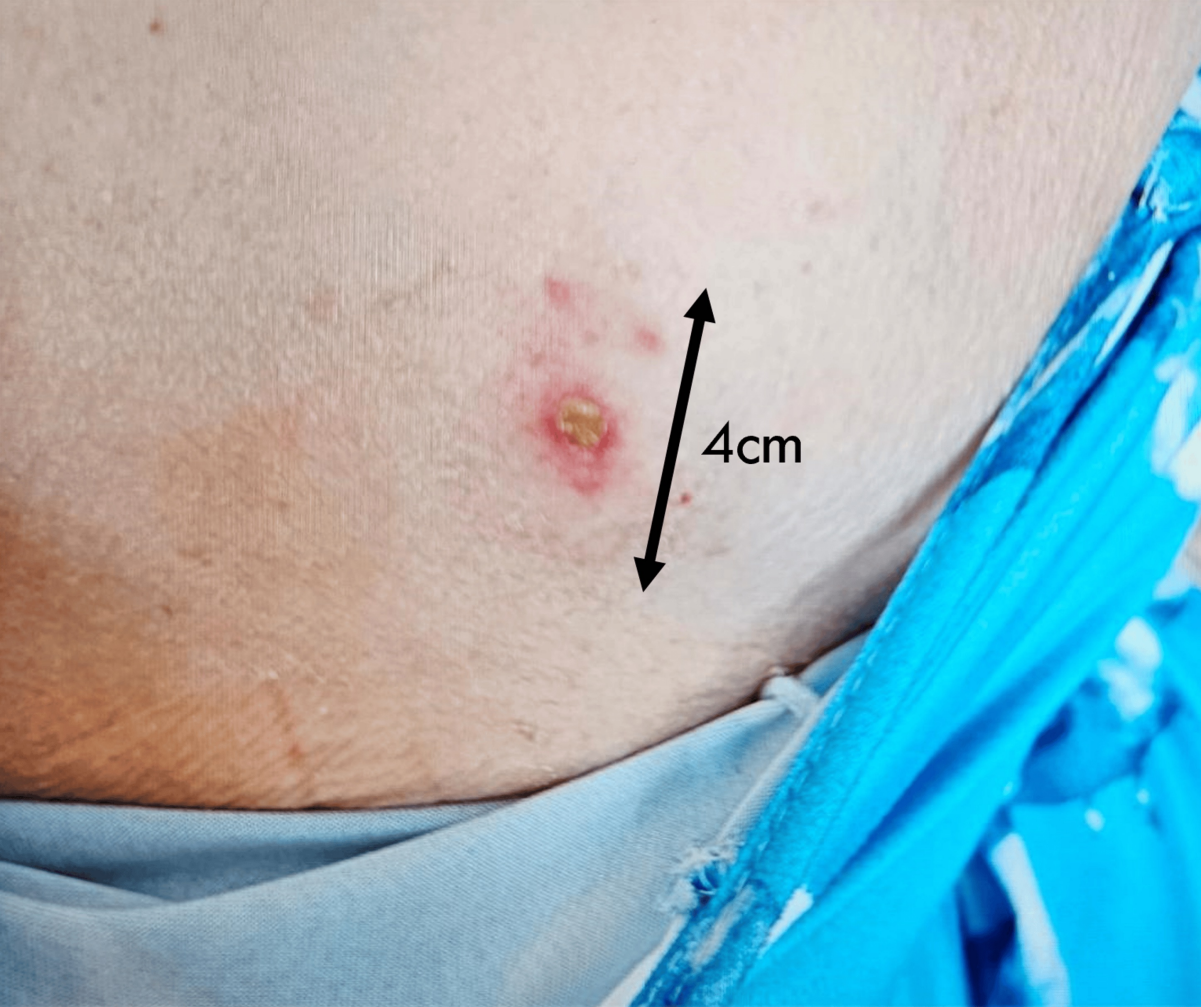
Chest wall wound on second presentation. The extent of induration is measured as shown

She was discharged after an inpatient stay of 14 days, with advice to continue oral amoxicillin three times a day and oral metronidazole 500 mg three times a day to complete a four-week course of combined antibiotics. A follow-up chest X-ray and an outpatient appointment were arranged. She was recovering well until a month after discharge. She began to notice swelling around the site where the drain was previously inserted. Over the next two days, the swelling gradually became more pronounced, the overlying skin became more erythematous, and finally, she noticed purulent discharge oozing from the drain site. She was seen in the respiratory follow-up clinic and was readmitted. A photo of the chest wall wound is shown in Figure 4.

At the time of readmission, the swelling and induration measured a maximum of 4 cm in diameter. Presenting symptoms included swelling, purulent discharge, and pain from the drain site. However, notably, she remained otherwise well. There was no cough, her shortness of breath had been improving since discharge, there were no fever-like symptoms, and there was no chest pain other than at the affected area. She did not report any confusion between the time of discharge and readmission. The systemic review was unremarkable.

**Figure 5:**
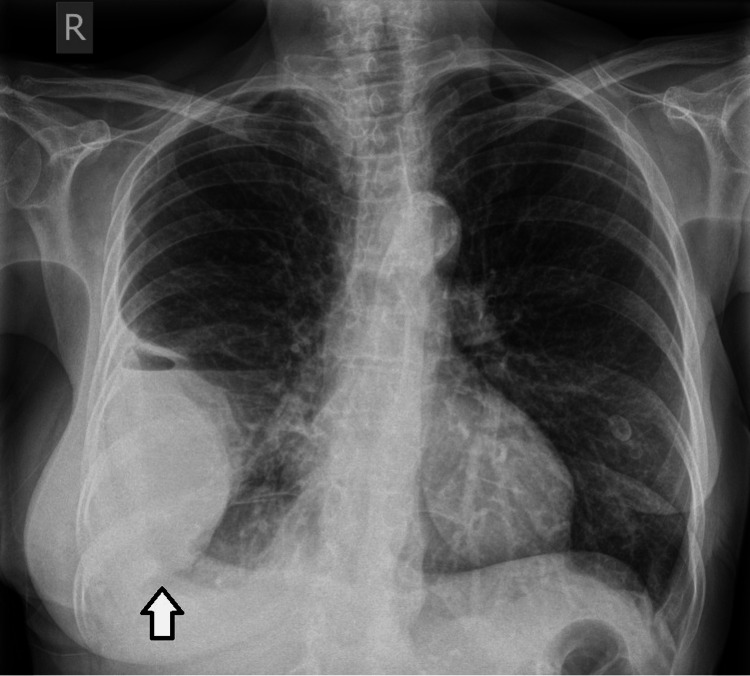
Chest X-ray on re-admission

**Figure 6:**
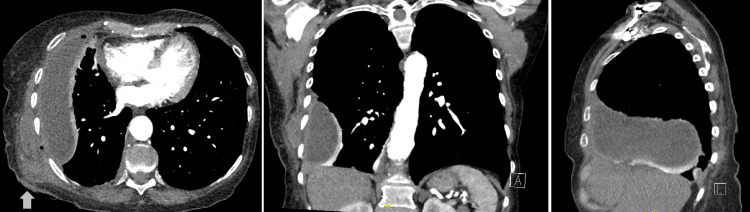
CT imaging on re-admission. Note the extension of fistulous tract to the subcutaneous tissue and skin

Her investigations on readmission showed a CRP of 32 mg/L and a white blood count of 12.3 (x10^9/L). A chest X-ray showed a new loculated collection peripherally in the right lower zone of the chest. A bedside thoracic ultrasound showed an organized multiloculated effusion with heterogeneous features, with an overlying abscess seen to be connecting with skin. To investigate further, a CT thorax was obtained, which showed a reduction in the overall size of the empyema and improved right lower lobe consolidation. However, the CT thorax also showed a new right posterior chest wall fluid collection measuring 4.1 cm × 4.6 cm × 3.0 cm. This was seen to directly communicate with the aforementioned pleural collection with a gas bubble seen in the intercostal space. This tract was shown to open to the skin. X-ray and CT imaging on re-admission are shown in Figures 5, 6.

**Figure 7:**
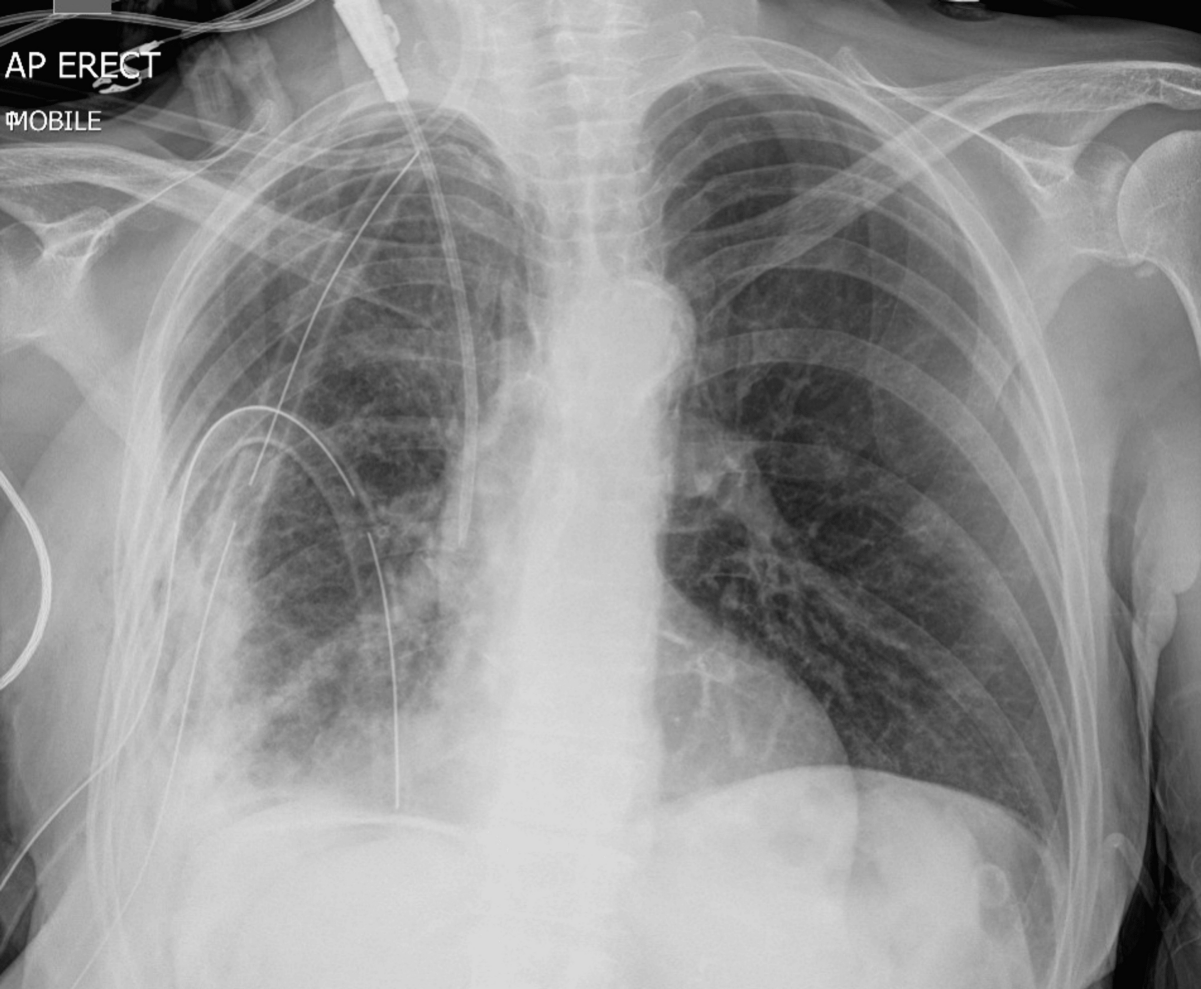
Chest X-ray following VATS. Note the presence of two large bore drains in the apical and basal regions of the right lung VATS: video-assisted thoracic surgery

A diagnosis of empyema necessitans was made. As per advice from specialists in infectious diseases, she was continued on oral amoxicillin 500 mg three times a day, with oral metronidazole 500 mg three times a day. She was transferred to the care of the cardiothoracic surgical team, who performed video-assisted thoracic surgery (VATS) for decortication and drainage. Intraoperatively, she was found to have an extensive abscess and soft tissue edema. The previous drain site was excised and debrided. During the procedure, the pleural space was found to be complex. A thick cortex right lower lobe was decorticated and washed out thoroughly with betadine. Two 32-french drains were placed (apically and basally), and the lung was seen to be visually inflating well. The previous drain site was felt to be unsuitable for direct closure; therefore, a vacuum dressing was also inserted intra-operatively. The chest X-ray images were taken immediately post-operatively (Figure 7).

Following surgical intervention, she remained in the ICU due to post-operative complications of low blood pressure, reduced urine output, and pre-renal AKI. She was treated for septic shock, requiring vasopressor support with noradrenaline. She also required one unit of blood transfusion post-operatively and fluid resuscitation with human albumin solution and normal saline. On discussion with the infectious diseases team, amoxicillin and metronidazole were stopped, and the antimicrobial regime was escalated to intravenous meropenem 1 g three times a day. By the third post-operative day, her urine output began to improve, and she was gradually weaned off noradrenaline. She was stepped down to the respiratory ward after five days of management in the intensive care unit.

**Figure 8:**
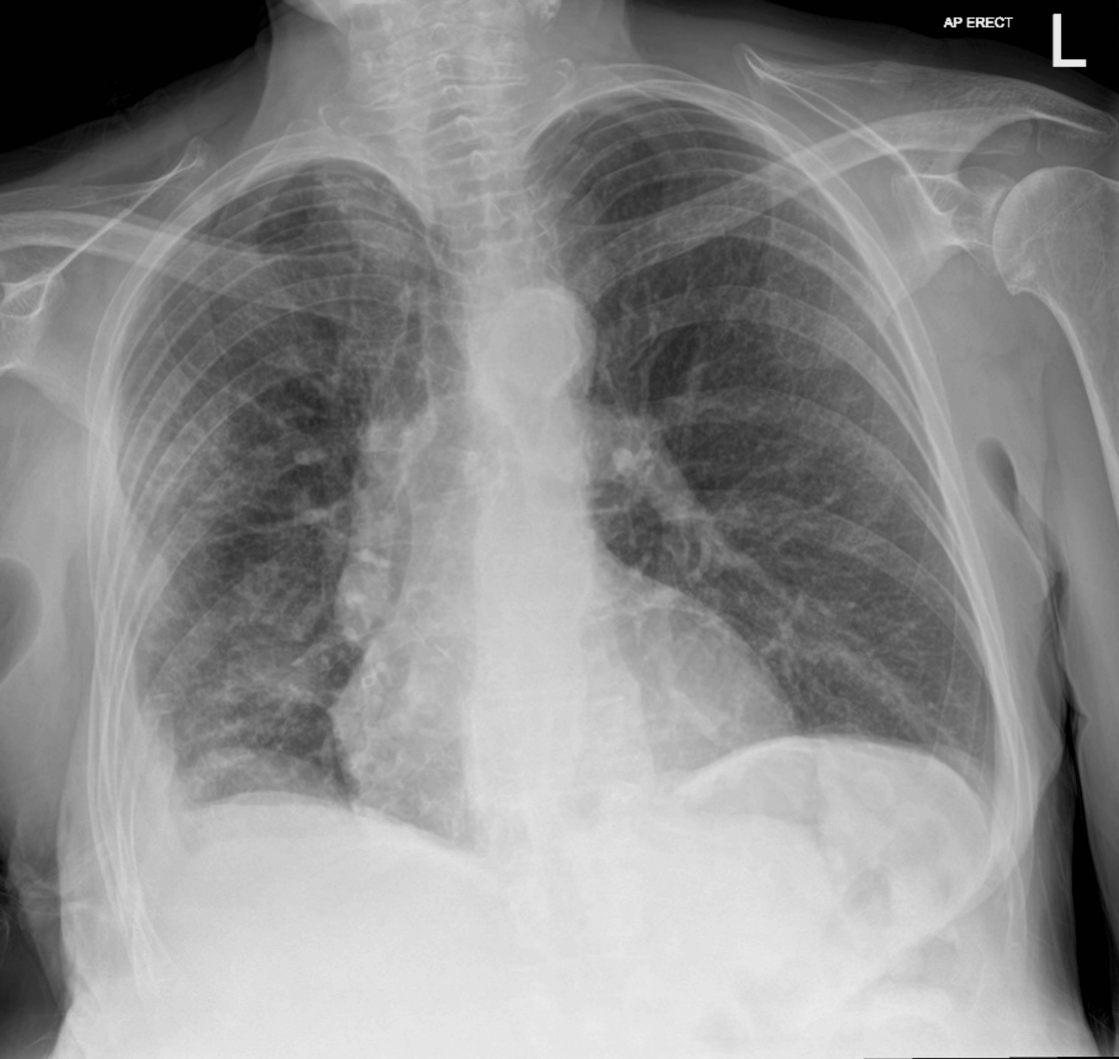
Chest X-ray two months after discharge

After completing a full-week course of meropenem, her antibiotics were reverted back to amoxicillin 500 mg three times a day and metronidazole 500 mg three times a day, with a view of completing four further weeks of combined antibiotics. She was subsequently discharged seven days after readmission. The apical drain and basal drains were removed on post-operative days 14 and 28, respectively. Wound swabs post-operatively continued to grow *Pseudomonas aeruginosa*, and she remains under follow-up. Figure 8 is the most recent chest X-ray done two months after discharge from her second admission.

## Discussion

Empyema necessitans is a rare complication of empyema, whereby intrathoracic pus communicates from within the pleural space to the soft tissue of the chest wall. In severe cases, this pus can be externalized to the skin. Empyema necessitans has historically been associated with inadequately treated pleural infections caused primarily by *M. tuberculosis* and *Actinomyces *spp. and is a clinical entity less frequently encountered these days due to the availability of timely and effective treatment [4]. Therefore, it must be emphasized that tuberculosis is an important differential for any case of empyema necessitans and should be ruled in or out with pleural testing. While there have been case reports on empyema necessitans occurring as a result of indwelling pleural catheters and chest drains, the literature surrounding empyema necessitans secondary to pleural intervention is scarce [3,6].

Empyema necessitans usually presents itself as localized mass and chest wall tenderness with overlying erythema, usually on a background of respiratory disease, with the most common location being the anterior chest wall where the lung is not as adherent to the chest wall [7]. Patients can often be septic on presentation. In the absence of systemic symptoms, the differential diagnosis for this would be broad, with the most common cause being a superficial drain site infection. While chest X-rays and thoracic ultrasounds can be quite helpful screening tools in visualizing empyema necessitans, chest CT is the best imaging modality to assess for the extent of disease outside of the thoracic cavity. The most common site of spread is the subcutaneous tissue of the chest wall, as was the case in our patient; empyema necessitans can also extend to other structures such as the pericardium, peritoneum, esophagus, or paravertebral regions [8].

Treatment for empyema necessitans includes a combination of antimicrobials guided by pleural fluid culture and sensitivities along with various surgical options. While a chest drain can be used as a first-line approach to treatment, surgical interventions like VATS decortication or open drainage are viable options, depending on factors like age, functional status, and extent of disease [9]. In our patient, VATS decortication was chosen as the preferred option to reduce the risk of new fistulization from chest drain insertion. VATS decortication has also been shown to reduce the length of hospital stay compared to chest drainage [9]. Relating to this, the latest European Respiratory Society (ERS) statement on pleural infections advocates early surgical referral with the aim of performing surgery within 10 days where indicated [10]. Despite a brief stay in the ICU following surgery, she managed to recover well and was discharged after a week.

As seen in literature, empyema necessitans can occur due to the virulence of the organism or is less commonly facilitated by previous pleural intervention. In our case, it was the site of chest drain insertion, which served as a fistulous tract for pus to spread from the pleural space, dissecting through the soft tissue outwards to the skin. Retrospectively, the chest X-ray done at the time of drain removal would suggest that the disease process started with a subcutaneous collection, with eventual extension toward the exterior. It would be helpful to obtain an early surgical review on the basis of these findings. However, given the clinical improvement at the time, the surgical risk would have outweighed the benefit of intervention in this case. This report emphasizes that despite being a rare condition, empyema necessitans should be considered in anyone presenting with chest wall swelling and pain, particularly in patients with a background of previous infections or thoracic intervention.

## Conclusions

This case serves as a good reminder about the complexity of various pleural interventional procedures, including relatively routine procedures such as the insertion of chest drains. Complications such as empyema necessitans are often not considered or mentioned while obtaining informed consent for these procedures. From our review of the literature on this topic, this is usually an early complication, seen while the chest drain/indwelling pleural catheter remains in place. This patient was treated in the first admission for empyema with the appropriate use of a chest drain for source control. She was then re-admitted a month after discharge, with empyema necessitans occurring weeks after the intervention and presenting with only very localized signs. It was interesting to note that our patient remained otherwise well in the period between the two admissions, and this is possibly due to the prolonged course of antibiotics she was receiving. Management for this patient demonstrates the importance of early intravenous antibiotic therapy, early chest drain insertion, and the importance of close monitoring, including regular inspection of drain sites for patients with empyema. When complicated by empyema necessitans, it highlights the benefit of early correspondence with surgical teams to ensure adequate source control. Our patient benefitted from early surgical intervention once the diagnosis of empyema necessitans was established. Whilst not systemically unwell when she presented on her second admission, her post-operative stay in the intensive care unit with septic shock underscores the potential of patients with this condition to rapidly deteriorate. This article adds to the relatively small number of articles describing this condition as a possible consequence of pleural interventions.
